# Modulatory effects of S1QEL on OxLDL-induced metabolic alterations and inflammatory responses in human monocytes

**DOI:** 10.1097/IN9.0000000000000090

**Published:** 2026-08-24

**Authors:** Negin Mosalmanzadeh, Rafael Moura Maurmann, Kierstin Davis, Brenda Landvoigt Schmitt, Liza Makowski, Brandt D. Pence

**Affiliations:** 1 College of Health Sciences, University of Memphis, Memphis, TN, USA; 2 Department of Medicine, Division of Hematology and Oncology, College of Medicine, University of Tennessee Health Science Center, Memphis, TN, USA; 3 Center for Cancer Research, University of Tennessee Health Science Center, Memphis, TN, USA; 4 Center for Nutraceutical and Dietary Supplement Research, University of Memphis, Memphis, TN, USA

**Keywords:** immunometabolism, glycolysis, inflammation, oxidative stress, reactive oxygen species, monocyte

## Abstract

**Background:**

Oxidized low-density lipoprotein (OxLDL) plays a key role in initiating monocyte activation, glycolytic reprogramming, and pro-inflammatory cytokine production—processes that contribute to aging and progression of age-related diseases such as atherosclerosis and steatohepatitis. Mitochondrial reactive oxygen species (ROS), particularly from complex I, are known modulators of these responses. This study aimed to evaluate the impact of OxLDL on the metabolic and inflammatory responses of primary human monocytes and determine whether co-treatment with site-1 Qo electron leak (S1QEL), a mitochondrial complex I-specific ROS suppressor, could mitigate these effects.

**Methods:**

Monocytes were isolated from healthy human donors and treated with OxLDL alone or in combination with S1QEL. Metabolic parameters including extracellular acidification rate (ECAR) and oxygen consumption rate (OCR) were measured using the Seahorse XF Analyzer. Pro-inflammatory gene expression (IL1B, CXCL8, IL6, TNF) was assessed using quantitative real-time polymerase chain reaction.

**Results:**

OxLDL treatment significantly increased ECAR, indicating enhanced glycolytic activity, without altering mitochondrial respiration (OCR). This metabolic shift was attenuated by S1QEL co-treatment. OxLDL also upregulated IL1B, CXCL8, and IL6 expression, which was significantly reduced by S1QEL. TNF expression remained unchanged across all conditions.

**Conclusions:**

S1QEL effectively suppresses both glycolytic reprogramming and pro-inflammatory cytokine expression induced by OxLDL in human monocytes. These findings underscore the role of mitochondrial complex I-derived ROS in monocyte activation and highlight S1QEL as a potential therapeutic agent for targeting inflammation in aging and several age-related diseases.

## 1. Introduction

Atherosclerosis, a predominant cause of cardiovascular diseases, arises from the buildup of lipids and fibrous elements within the arteries, a process centrally involving oxidized low-density lipoprotein (OxLDL) ^[1]^. OxLDL is instrumental in the development of atheromatous plaques, complex arterial wall structures that are the hallmark of atherosclerosis. Infiltrating monocytes in the plaque accumulate OxLDL, evolving into foam cells ^[2]^. The formation of foam cells marks a critical juncture in the progression of atherosclerosis, contributing significantly to the growth and instability of arterial plaques ^[3]^, and foam cells increase the risk of plaque rupture, which can lead to myocardial infarction or stroke ^[4]^.

OxLDL, apart from being a lipid-carrying molecule, acts as a pro-inflammatory stimulus, activating various signaling pathways within monocytes and macrophages. This activation leads to the production of inflammatory cytokines and chemokines, which further recruit immune cells to the site, exacerbating the inflammatory response. When activated by inflammation, myeloid cells undergo a significant metabolic shift towards glycolysis ^[5]^. Furthermore, mitochondrial dynamics and oxidative stress play essential roles in these inflammatory responses. A notable contributor to mitochondrial oxidative stress is the production of reactive oxygen species (ROS) from mitochondrial complex I ^[6]^.

Site-1 Qo electron leak (S1QEL), on the other hand, is a compound that specifically targets the quinone-reaction site within mitochondrial complex I during reverse electron transfer ^[7]^, aiming to alleviate oxidative stress and its downstream effects. Its role in modulating metabolic pathways also makes S1QEL a compound of significant interest in the context of diseases like atherosclerosis, where metabolic dysfunction plays a central role. By improving mitochondrial efficiency and reducing oxidative stress, S1QEL could contribute to ameliorating the metabolic and inflammatory disturbances seen in atherosclerotic plaques.

The capabilities of S1QELs in modulating mitochondrial function and reducing oxidative stress, especially through their interactions with mitochondrial complex I, are not fully understood. The range of effects these compounds may have under cellular stress conditions induced by OxLDL remains a largely untapped area of research. Unraveling the complexities of how S1QELs affect various cellular functions and responses to pathological stimuli is crucial, considering their potential therapeutic applications in a wide array of cell types and diseases.

In light of these significant gaps, particularly concerning the role of S1QEL in monocyte function under atherosclerotic stressors like OxLDL, this study is designed to make a substantial contribution to the field. The primary objective is to investigate the potential of S1QEL in mitigating the harmful effects of OxLDL on monocyte function, encompassing a meticulous examination of metabolic alterations, inflammatory cytokine production, foam cell formation, mitochondrial morphological changes, and mitochondrial ROS (mtROS) production. This comprehensive investigation seeks to illuminate the nuanced pathways through which monocytes contribute to atherosclerosis, not only aiming to deepen our understanding of mitochondrial dynamics in monocyte function but also to explore novel therapeutic avenues. By uncovering the multifaceted roles of these compounds in monocyte behavior and their overall impact on the progression of atherosclerosis, the research promises to pave the way for innovative therapeutic strategies targeting these critical cellular processes.

## 2. Materials and methods

### 2.1 Participants

Healthy, nonobese young adults aged 18 to 35 were recruited for the study. Participants with any disease related to inflammation, immune dysfunction, or metabolic dysfunction, or those on medications affecting inflammation or metabolism, were excluded. All participants signed a written informed consent before participation. The study received approval from the Institutional Review Board of the University of Memphis under protocol PRO-FY2021-476 (approval date: June 24, 2022). Both male and female participants were recruited without regard to race or other physiological factors. Previous studies showed no differential effects of race and sex on ex vivo monocyte responses to lipopolysaccharide, and similar results were anticipated for OxLDL in macrophages. However, to address the importance of sex and race as biological variables, sample sizes were planned to be increased if initial findings indicated their potential influence on responses to OxLDL or S1QEL. Participants were required to visit the lab up to six times, arriving in a fasted state for blood collection for monocyte isolation. Blood (8–16 mL) was collected by venipuncture into ethylenediaminetetraacetic acid (EDTA) vacutainer tubes.

### 2.2 Monocyte isolation

Monocytes were isolated from blood samples using a negative selection method, which involved magnetic bead separation employing EasySep Direct Human Monocyte Isolation Kits (STEMCELL Technologies, Vancouver, BC, Canada). This technique ensured the attainment of a highly pure monocyte population with minimal contamination from other cell types. In previous studies, our methodology consistently yielded 90% to 95% pure and >90% viable monocyte populations, with no observed differences attributable to race, sex, or age ^[8]^.

### 2.3 Metabolic activation assays

Isolated monocytes were seeded at 1.5 × 10^5^ cells/well in Seahorse XFp 8-well plates (Agilent, Santa Clara, CA, USA), using Seahorse XF media enriched with 10 mM d-glucose and 2 mM L-glutamine, and total well volume was adjusted to 180 μL with supplemented media. S1QEL (Sigma, St. Louis, MO, USA) was prepared at a stock concentration of 1 mM and stored at −20 °C until use. S1QEL was diluted to 50 μM in supplemented Seahorse media immediately before use. For Seahorse assays, 20 μL of supplemented media or S1QEL was added to wells for a total volume of 200 μL. Resulting final concentration of S1QEL was 5 μM. The plate was centrifuged and incubated at 37 °C in a non-CO_2_ incubator for 1 hour. OxLDL solution (2.5 mg/mL) or media was added to injection ports of the Seahorse XFp cartridge to create three groups of control (media-only), OxLDL, and OxLDL plus S1QEL. Monocytes were exposed to OxLDL for 2 hours before downstream metabolic and inflammatory analyses. Serial measurements of oxygen consumption rate (OCR) and extracellular acidification rate (ECAR) were recorded using a Seahorse XFp analyzer (Agilent, Santa Clara, CA, USA) for 30 minutes before and 3 hours following injection of OxLDL. Postanalysis, cell counts were conducted by photomicroscopy. Cells were subsequently lysed with TRIzol (Thermo Fisher, Waltham, MA, USA) and stored at −80 °C for cytokine gene expression analysis as previously described ^[9,10]^. For initial experiments without S1QEL, the same procedures were followed, with no S1QEL pretreatment.

### 2.4 Inflammatory cytokine gene expression

RNA Extraction: RNA was extracted from primary monocyte lysates using the Trizol method. Lysates were thawed, mixed with 1-Bromo-3-chloropropane, and centrifuged to separate the RNA-containing phase. The RNA was precipitated with isopropanol and glycogen, washed with 75% ethanol, and air-dried. This RNA was dissolved in RNase-free water, quantified, and stored at −80 °C.

Quantitative real-time polymerase chain reaction R (qRT-PCR): Quantified RNA was reverse transcribed to cDNA using the high-capacity cDNA Kit (Thermo Fisher, Waltham, MA, USA). The cDNA synthesis involved components like RT Buffer, dNTP Mix, RT random primers, MultiScribe RT, RNase inhibitor, and nuclease-free H_2_O. This cDNA was used in qPCR reactions with the TaqMan Fast Adv. Master Mix and gene-specific TaqMan gene expression assays. Amplification conditions included an initial hold at 50 °C, polymerase activation at 95 °C, followed by 40 cycles of PCR. Gene expression levels were analyzed using the comparative Ct method, with Beta-actin (ACTB) as a reference gene.

### 2.5 ROS production assessment

Isolated monocytes were plated at 2.5 × 10^5^ per well in an 8-well plate. Cells were stimulated with CHO-SFM II macrophage media (Gibco, Waltham, MA, USA) or 1 mg/mL OxLDL. After a 2.5-hour incubation, cells were washed and stained with MitoSOX Green (10 µM), a mitochondrial superoxide indicator, for 30 minutes before imaging. Cells were imaged at 40× on a fluorescence microscope (EVOS M7000, Thermo Fisher, Waltham, MA, USA). Individual cells were quantified for fluorescence intensity in ImageJ (NIH, Bethesda, MD, USA) and background-corrected against adjacent noncell areas. An identical experiment was performed with 1-hour pretreatment of cells with 5 μM S1QEL or media, followed by stimulation with OxLDL, to assess the effect of S1QEL on OxLDL-mediated ROS production.

### 2.6 Statistical analysis

To compare two treatments, paired Student’s *T* tests were employed for normally distributed data and Wilcoxon tests as a nonparametric alternative. For comparisons involving three or more treatments, repeated measures analysis of variance (parametric) or Friedman tests (nonparametric) were utilized, along with Holm–Bonferroni post-hoc testing for multiple testing-adjusted mean separation. The significance level was set at α = 0.05. Statistical testing was performed using R 4.4.1 (R Core Team, Vienna, AUT).

### 2.7 Ethical approval

All participants signed a written informed consent before participation. The study received approval from the Institutional Review Board of the University of Memphis under protocol PRO-FY2021-476 (approval date: June 24, 2022). This research was in line with the Declaration of Helsinki.

## 3. Results

### 3.1 OxLDL reprograms monocyte metabolism

**Figure 1.**
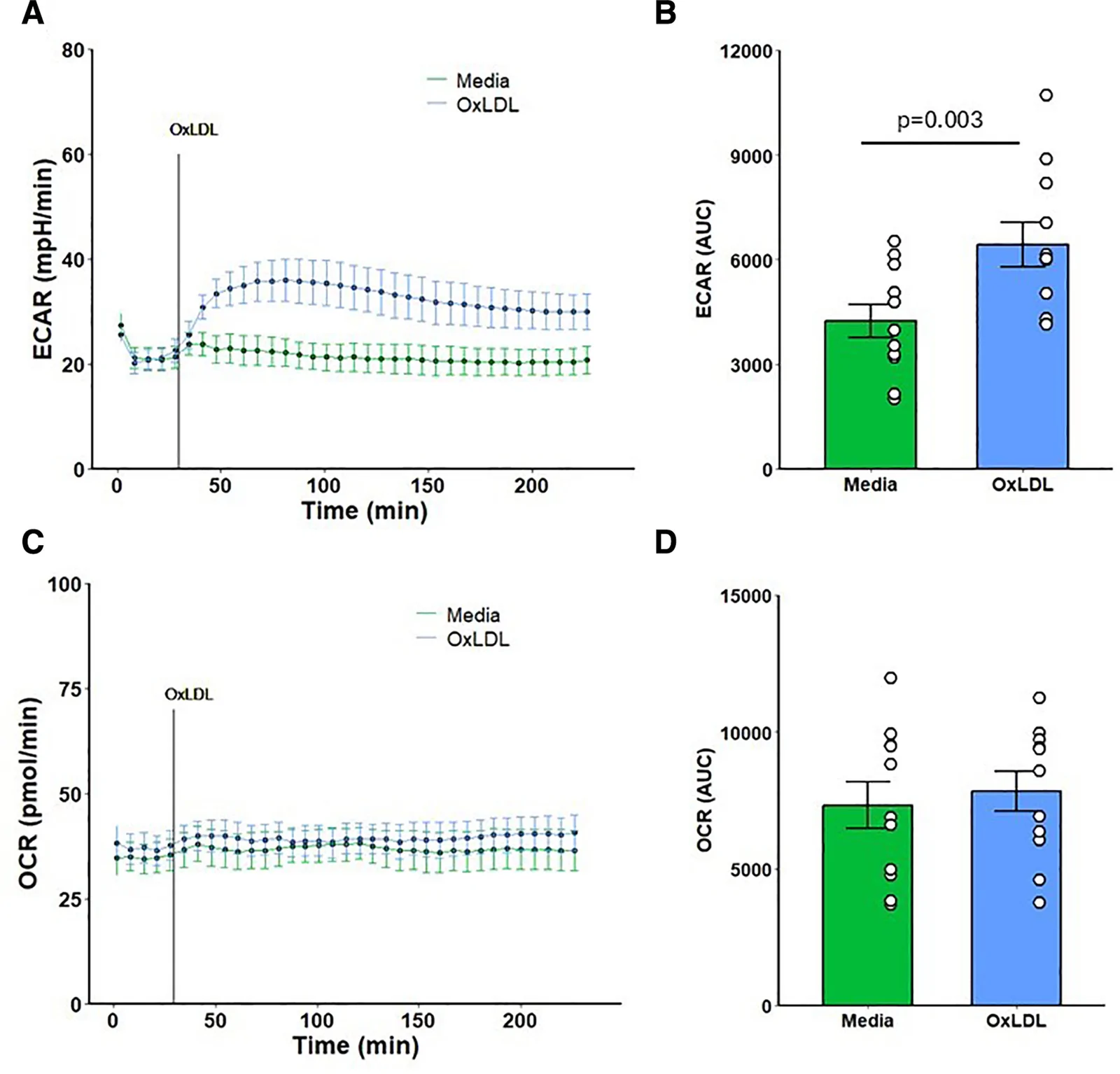
(A) ECAR measured over time in monocytes treated with media or OxLDL. (B) AUC of the ECAR response to media or OxLDL. (C) OCR measured over time in monocytes treated with media or OxLDL. (D) AUC of the OCR response to media or OxLDL. AUC, area under the curve; ECAR, extracellular acidification rate; OCR, oxygen consumption rate; OxLDL, oxidized low-density lipoprotein.

Treatment of monocytes with OxLDL lead to an increase in acidification rate compared with the media-only control (Figure 1A). Statistical analysis of the area under the curve (AUC) for ECAR revealed a significant increase in glycolytic activity in OxLDL-treated cells compared with control (*P* = 0.003, Figure 1B). For OCR, an indicator of mitochondrial respiration, no significant difference was observed over time across all treatment conditions (Figure 1C). The AUC analysis for OCR also indicated no significant changes, suggesting that OxLDL did not substantially affect mitochondrial respiration under the conditions tested (Figure 1D).

**Figure 2.**
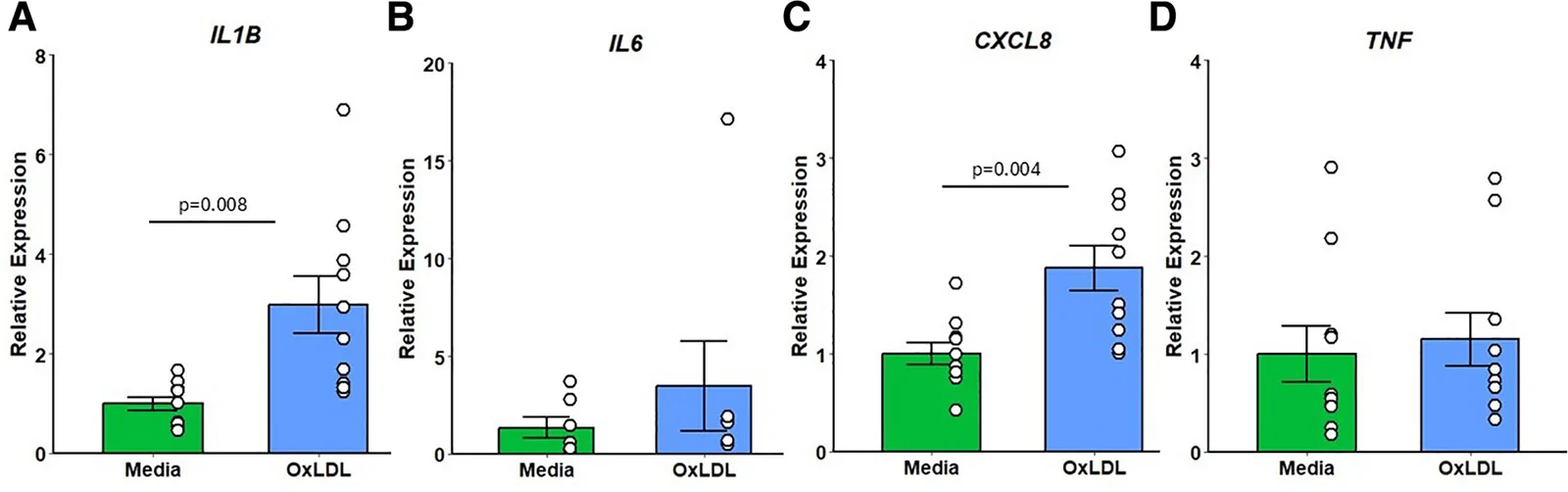
Relative expression levels of IL1B, CXCL8, IL6, and TNF in monocytes treated with media or OxLDL. Statistical significance is indicated on the bar graphs.

Additionally, monocyte expression of IL1B and CXCL8 was significantly elevated in the presence of OxLDL (*P* = 0.008 and *P* = 0.004, respectively, Figure 2A,C). IL6 and TNF expression levels did not significantly change with OxLDL treatment (Figure 2B,D).

### 3.2 Reactive oxygen species production

**Figure 3.**
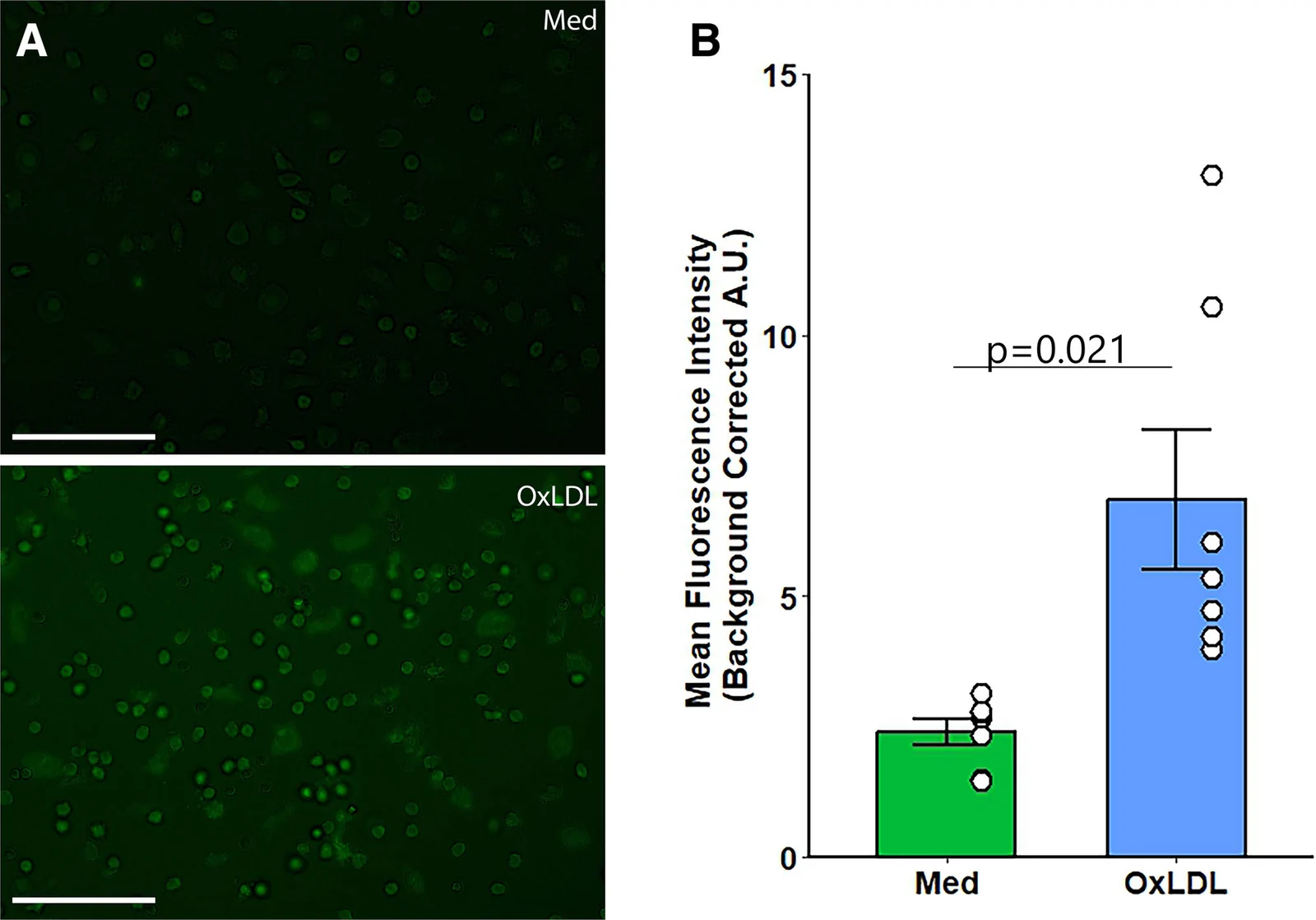
(A) Representative 40× fluorescence microscopy images of monocytes treated with OxLDL and media, showing differences in fluorescence intensity as an indicator of mtROS levels. (B) The bar graph quantifies mean fluorescence intensity (background-corrected arbitrary units, A.U.) in the different treatment groups. Statistical significance indicated (*P* = 0.021). Scale bar = 70 µm. mtROS, mitochondrial reactive oxygen species; OxLDL, oxidized low-density lipoprotein.

We assessed the production of mtROS in the presence or absence of OxLDL. The assessment of mtROS production revealed a notable difference between monocytes cultured in standard media and those treated with OxLDL (Figure 3). Fluorescence microscopy images showed a minimal fluorescence signal in the control group, indicating low levels of mtROS. In contrast, cells treated with OxLDL exhibited a significant increase in fluorescence intensity, indicative of elevated mtROS levels (*P* = 0.021).

### 3.3 Effects of S1QEL

**Figure 4.**
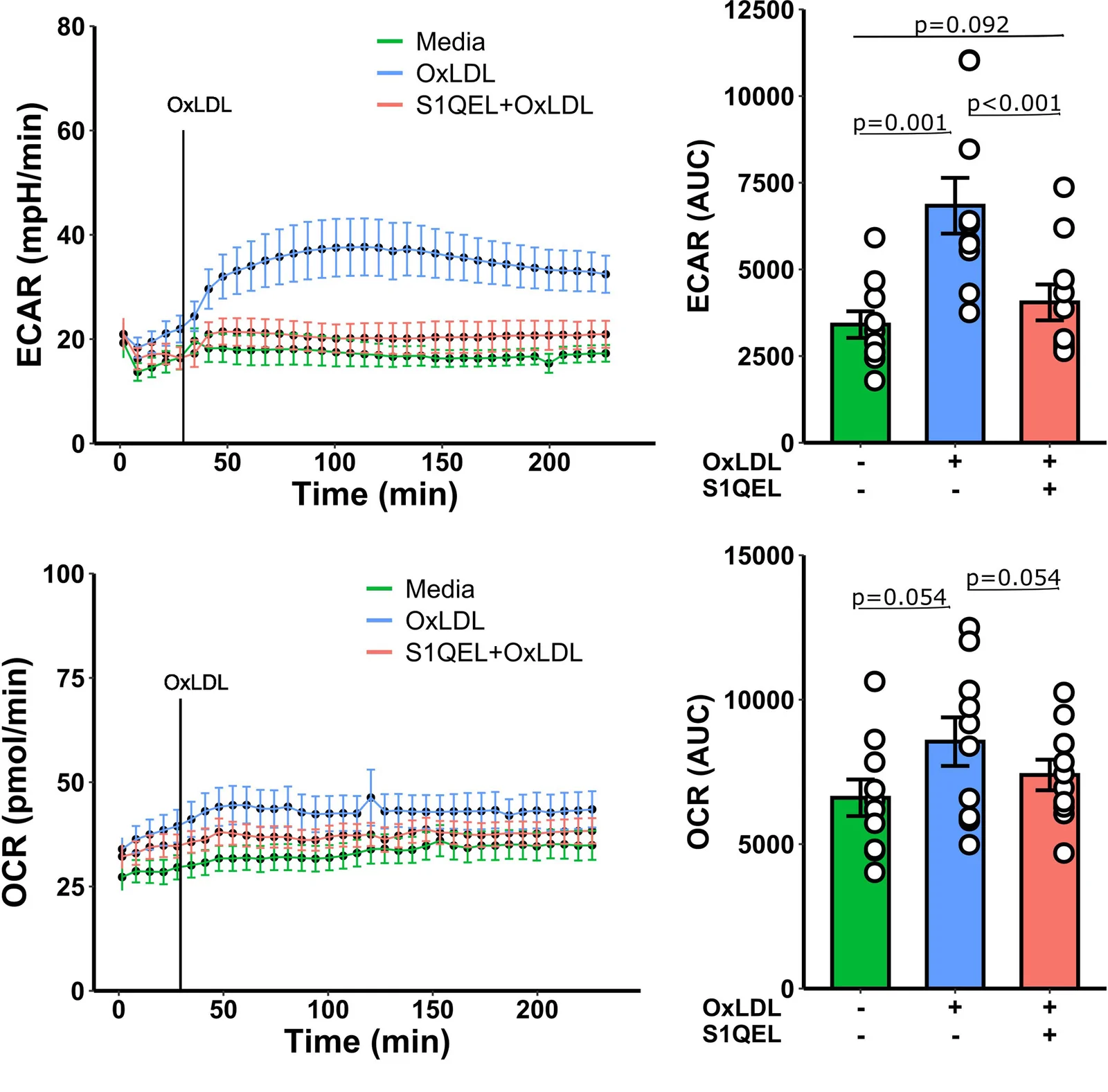
(A) ECAR measured over time in monocytes treated with media, OxLDL, or S1QEL+OxLDL. (B) The bar graph represents the AUC for ECAR with statistical significance indicated (*P* = 0.001 for OxLDL vs media, *P* < 0.001 for S1QEL+OxLDL vs OxLDL). (C) OCR measured over time in monocytes treated with media, OxLDL, or S1QEL+OxLDL. (D) The bar graph represents the AUC for OCR. AUC, area under the curve; ECAR, extracellular acidification rate; OCR, oxygen consumption rate; OxLDL, oxidized low-density lipoprotein; S1QEL, site-1 Qo electron leak.

Given that OxLDL increased mtROS, we examined the ability of S1QEL, a selective complex I-associated ROS inhibitor, to suppress glycolytic and inflammatory responses to OxLDL in monocytes. In general, S1QEL reversed OxLDL-mediated effects on metabolic reprogramming and inflammation. OxLDL increased ECAR, which was significantly reduced in the presence of S1QEL (*P* < 0.001), indicating a regulatory effect of S1QEL on glycolysis (Figure 4A,B). The OCR did not significantly change with OxLDL or S1QEL treatment, suggesting that mitochondrial oxidative capacity was maintained across conditions (Figure 4C,D).

**Figure 5.**
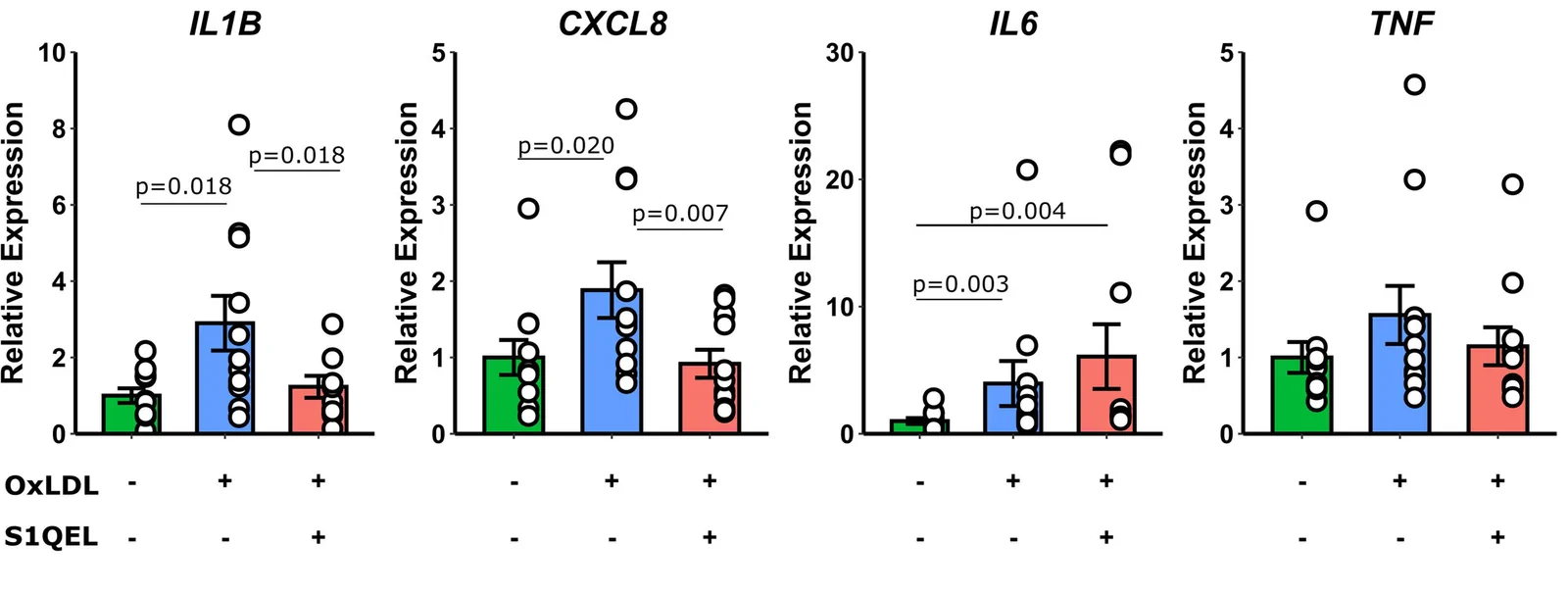
Relative expression levels of IL1B, CXCL8, IL6, and TNF in monocytes treated with media, OxLDL, or S1QEL+OxLDL. Statistical significance is indicated on the bar graphs. OxLDL, oxidized low-density lipoprotein; S1QEL, site-1 Qo electron leak.

Additionally, treatment with OxLDL significantly increased the expression of IL1B, CXCL8, and IL6 (*P* = 0.018, *P* = 0.020, and *P* = 0.003, respectively, Figure 5A–C). Co-treatment with S1QEL and OxLDL significantly reduced the expression of these cytokines (IL1B: *P* = 0.018, CXCL8: *P* = 0.007, IL6: *P* = 0.004). The expression of TNF remained unaltered across all conditions (Figure 5D).

**Figure 6.**
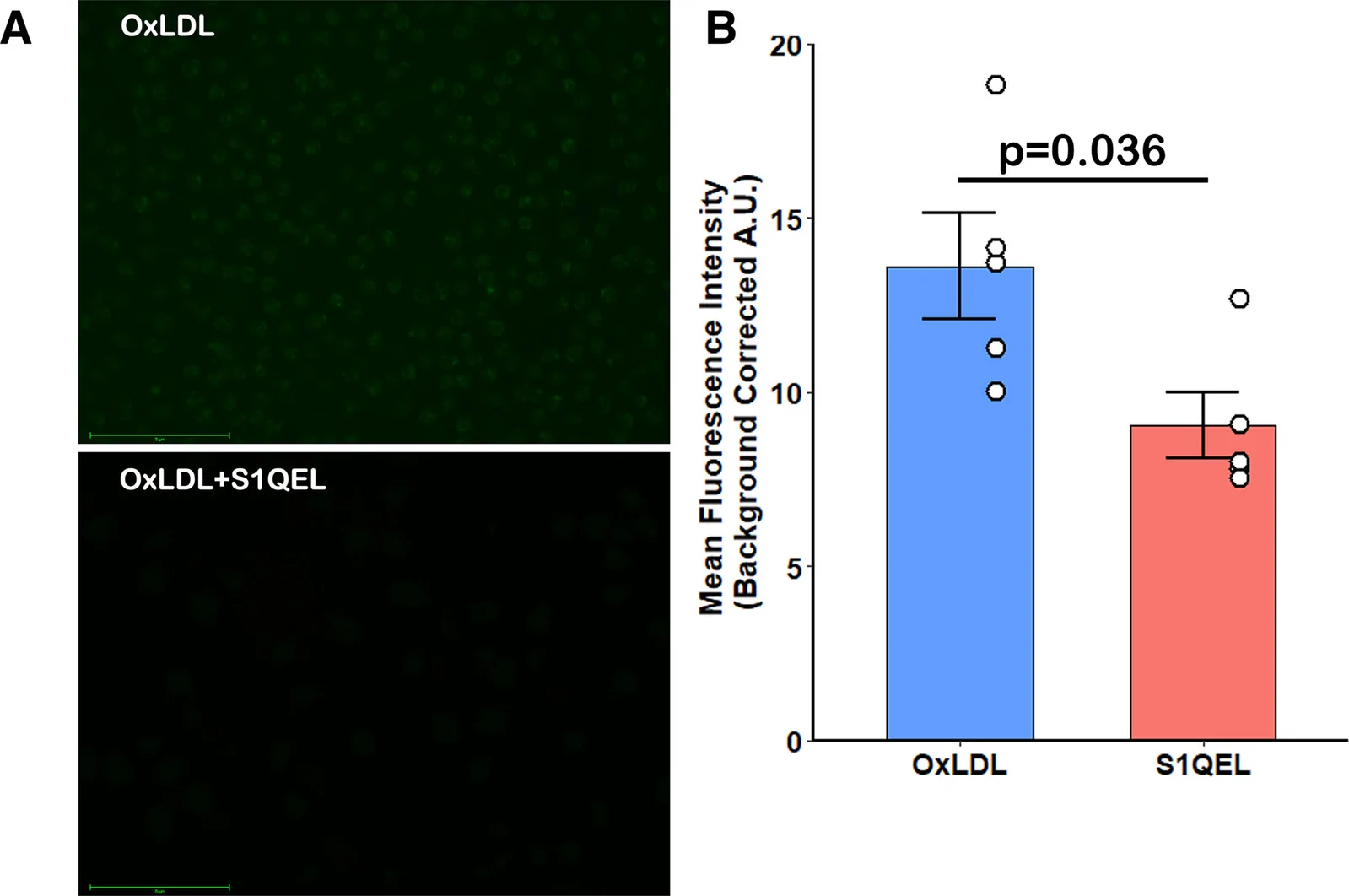
(A) Representative 40× fluorescence microscopy images of monocytes treated with OxLDL and pretreated with S1QEL or media, showing differences in fluorescence intensity as an indicator of mtROS levels. (B) The bar graph quantifies mean fluorescence intensity (background-corrected arbitrary units, A.U.) in the different treatment groups. Statistical significance indicated (*P* = 0.036). Scale bar = 70 µm. mtROS, mitochondrial reactive oxygen species; OxLDL, oxidized low-density lipoprotein; S1QEL, site-1 Qo electron leak.

Finally, we verified that S1QEL suppresses mtROS production in monocytes. Cells pretreated with S1QEL had suppressed OxLDL-stimulated ROS production compared to media-treated OxLDL-stimulated controls (Figure 6).

## 4. Discussion

In this study, we investigated the impact of OxLDL on monocyte metabolism and inflammatory gene expression, and whether these effects could be mitigated by co-treatment with S1QEL. Our findings show that exposure to OxLDL led to a significant increase in glycolytic activity, as evidenced by elevated ECAR, without a concurrent change in mitochondrial respiration (OCR). These results are consistent with a metabolic shift toward glycolysis, a hallmark of inflammatory activation in monocytes ^[11]^.

Notably, similar glycolytic remodeling has been reported in “trained immunity” paradigms where human primary monocytes exposed to oxLDL display increased ECAR (basal and/or maximal glycolysis), supporting the idea that oxLDL can prime myeloid cells toward a sustained, glycolysis-forward state ^[12]^. In this context, our acute (hours-scale) ECAR elevation without a detectable OCR change may reflect an early bioenergetic “decoupling", where glycolytic flux rises rapidly to support inflammatory signaling and biosynthetic demand while mitochondrial oxidative capacity remains grossly intact over the same window ^[13]^. This pattern also aligns with the concept that inflammatory stimulation can increase lactate production and glycolytic throughput even when OxPhos is not overtly suppressed, depending on stimulus strength, timing, and donor-to-donor variability.

At the same time, the oxLDL literature is not fully uniform: some macrophage-focused studies report oxLDL accumulation attenuating glycolysis and dampening inflammatory programs, suggesting context-dependent effects driven by factors such as degree of oxidation, lipid loading, exposure duration, and differentiation state (monocyte vs macrophage/foam cell) ^[14]^. Relatedly, some work indicates that highly oxidized LDL may only modestly activate primary monocytes under certain conditions ^[15]^. These apparent discrepancies emphasize that “oxLDL” is not a single entity—differences in preparation/oxidation chemistry and experimental timing can shift the balance between pro-glycolytic inflammatory activation versus lipid-stress programs (eg, antioxidant/nuclear factor erythroid 2-related factor 2-biased responses) ^[14]^.

S1QEL co-treatment significantly reduced OxLDL-induced glycolytic activation, suggesting that ROS derived from mitochondrial complex I may contribute to glycolytic reprogramming by influencing redox-sensitive signaling pathways. Importantly, S1QEL did not affect OCR, indicating that its modulatory role is specific to glycolytic flux rather than general mitochondrial respiration.

Mechanistically, this “selective” phenotype is consistent with how S1QEL-class compounds are described to suppress superoxide/H_2_O_2_ generation from the complex I Q-site (site I_Q) without broadly inhibiting forward electron transport, which would be expected to preserve bulk respiration (OCR) while dampening ROS-dependent signaling ^[16,17]^. Recent work supports that site I_Q ROS can occur in cells during forward electron transfer as well as RET-like conditions and remains S1QEL-sensitive, providing a plausible biochemical basis for our observation that inflammatory/metabolic outputs can be reduced without collapsing mitochondrial oxidative capacity ^[16]^. In other immune contexts, inhibition of the complex I IQ site with S1QEL.1 has been shown to reduce ROS and dampen activation markers in stimulated CD8^+^ T cells, supporting the broader principle that complex I-derived ROS can act as an immunometabolic “gain control” signal ^[18]^.

In parallel, we observed that OxLDL significantly upregulated the expression of key pro-inflammatory cytokines IL1B, CXCL8, and IL6, confirming its role in promoting inflammatory activation in monocytes. S1QEL treatment attenuated the expression of these cytokines, demonstrating its potential to suppress inflammation. Notably, TNF expression remained unchanged, suggesting that the inflammatory response mediated by OxLDL may involve selective signaling mechanisms.

The cytokine pattern we observed (IL1B/CXCL8/IL6 responsive, TNF comparatively stable) is biologically plausible given that oxLDL-triggered programs can preferentially engage inflammasome-associated and chemotactic axes, while TNF can be highly timing- and context-sensitive (often peaking early and transiently, or requiring co-stimulation) ^[19]^. A practical implication is that TNF “nonresponse” here may reflect kinetics (sampling time point), stimulus specificity, or donor heterogeneity rather than absence of inflammatory activation per se—especially since ECAR and multiple cytokine transcripts shift robustly. Future time-course sampling could help distinguish “true non-responsiveness” from a missed TNF peak.

These results collectively indicate that S1QEL effectively counteracts both metabolic and inflammatory alterations induced by OxLDL in monocytes. Given the central role of monocyte activation in atherogenesis, targeting mitochondrial complex I-derived ROS could represent a novel anti-inflammatory strategy in cardiovascular disease. To our knowledge, this is one of the first studies demonstrating the ability of S1QEL to modulate both metabolic and inflammatory pathways in primary human monocytes.

Our mtROS data provide an important bridge between OxLDL exposure and downstream immunometabolic remodeling, consistent with broader frameworks in which mitochondrial ROS act as signaling intermediates that tune innate immune activation and inflammatory gene programs ^[13,20]^. In line with this, studies manipulating complex I function in myeloid cells show that complex I disruption can increase mtROS and promote a glycolysis-forward metabolic phenotype that can “prime” heightened inflammatory responses under additional stimulation—supporting the idea that complex I redox state is upstream of immunometabolic polarization ^[21]^. Thus, our results fit a model where OxLDL elevates mtROS, mtROS contributes to glycolytic and cytokine reprogramming, and S1QEL interrupts this axis by suppressing site I_Q electron leak without broadly impairing oxidative phosphorylation ^[16,17]^.

Despite the promising findings, our study has limitations. Being conducted in vitro, the results may not fully capture the complexity of in vivo systems, where cell–cell interactions and systemic factors play a significant role. Future studies involving animal models or clinical samples are necessary to validate these findings and explore the long-term effects of S1QEL treatment. Furthermore, mechanistic studies are warranted to delineate the exact pathways through which complex I-derived ROS mediate metabolic and inflammatory changes in monocytes.

## 5. Conclusions

Our findings demonstrate that OxLDL promotes glycolytic reprogramming and upregulates pro-inflammatory cytokine expression in primary human monocytes. Co-treatment with S1QEL, a mitochondrial complex I-specific ROS suppressor, significantly attenuated both metabolic and inflammatory responses without affecting mitochondrial respiration. These results highlight the central role of mitochondrial ROS, particularly from complex I, in mediating OxLDL-induced monocyte activation.

By modulating both metabolism and inflammation, S1QEL emerges as a promising candidate for therapeutic strategies targeting monocyte-driven inflammation in atherosclerosis. Further in vivo studies are warranted to validate its efficacy and explore its long-term safety and applicability in clinical settings. This study provides new insights into the intersection of mitochondrial function, oxidative stress, and immune cell metabolism in cardiovascular disease.

## Author contributions

Conceptualization: BP, LM; Formal analysis: NM, BP; Funding acquisition: BP; Investigation: BP, NM, RMM, KD, BLS; Methodology: BP, NM; Project administration and supervision: BP, Writing-original draft: NM, Writing-review and editing: NM, BP, RMM, KD, BLS, LM. All authors have read and approved the manuscript.

## Conflicts of interest

The authors declare that they have no conflicts of interest

## Funding

This project was funded by the American Heart Association (19TPA34910132) and National Institute on Aing (R15AG078906) grants to BDP. LM is a co-investigator on AHA 19TPA34910132. BLS is supported by National Cancer Institute grant U01CA272541 to LM.

## Acknowledgements

The authors thank the study participants for their contributions.

## References

[1] Kita T Kume N Minami M. Role of oxidized LDL in atherosclerosis. Ann N Y Acad Sci. 2001;947(1):199-205; discussion 205. doi: 10.1111/J.1749-6632.2001.TB03941.X. 11795267 10.1111/j.1749-6632.2001.tb03941.x

[2] Boullier A Bird DA Chang MK. Scavenger receptors, oxidized LDL, and atherosclerosis. Ann N Y Acad Sci. 2001;947:214-22; discussion 222. doi: 10.1111/J.1749-6632.2001.TB03943.X. 11795269 10.1111/j.1749-6632.2001.tb03943.x

[3] Guyton JR Klemp KF. Development of the lipid-rich core in human atherosclerosis. Arterioscler Thromb Vasc Biol. 1996;16(1):4-11. doi: 10.1161/01.ATV.16.1.4. 8548424 10.1161/01.atv.16.1.4

[4] Bentzon JF Otsuka F Virmani R. Mechanisms of plaque formation and rupture. Circ Res. 2014;114(12):1852-66. doi: 10.1161/CIRCRESAHA.114.302721. 24902970 10.1161/CIRCRESAHA.114.302721

[5] Shi C Pamer EG. Monocyte recruitment during infection and inflammation. Nat Rev Immunol. 2011;11(11):762-74. doi: 10.1038/NRI3070. 21984070 10.1038/nri3070 PMC3947780

[6] Weinberg SE Chandel NS. Mitochondria reactive oxygen species signaling-dependent immune responses in macrophages and T cells. Immunity. 2025;58(8):1904-21. doi: 10.1016/J.IMMUNI.2025.07.012. 40763730 10.1016/j.immuni.2025.07.012 PMC12371701

[7] Brand MD Goncalves RLS Orr AL. Suppressors of superoxide-H2O2 production at Site IQ of mitochondrial complex I protect against stem cell hyperplasia and ischemia-reperfusion injury. Cell Metab. 2016;24(4):582-92. doi: 10.1016/j.cmet.2016.08.012. 27667666 10.1016/j.cmet.2016.08.012 PMC5061631

[8] Pence BD Yarbro JR. Aging impairs mitochondrial respiratory capacity in classical monocytes. Exp Gerontol. 2018;108:112-7. doi: 10.1016/j.exger.2018.04.008. 29655929 10.1016/j.exger.2018.04.008

[9] Yarbro JR Pence BD. Classical monocytes from older adults maintain capacity for metabolic compensation during glucose deprivation and lipopolysaccharide stimulation. Mech Ageing Dev. 2019;183:111146. doi: 10.1016/j.mad.2019.111146. 31493436 10.1016/j.mad.2019.111146 PMC7027594

[10] Pence BD Yarbro JR. Classical monocytes maintain ex vivo glycolytic metabolism and early but not later inflammatory responses in older adults. Immun Ageing. 2019;16:3. doi: 10.1186/s12979-019-0143-1. 30700992 10.1186/s12979-019-0143-1 PMC6348080

[11] Soto-Heredero G Gómez de las Heras MM Gabandé-Rodríguez E. Glycolysis - a key player in the inflammatory response. FEBS J. 2020;287(16):3350-69. doi: 10.1111/FEBS.15327. 32255251 10.1111/febs.15327 PMC7496292

[12] Keating ST Groh L Thiem K. Rewiring of glucose metabolism defines trained immunity induced by oxidized low-density lipoprotein. J Mol Med (Berl). 2020;98(6):819-31. doi: 10.1007/s00109-020-01915-w. 32350546 10.1007/s00109-020-01915-w PMC7297856

[13] Cortés M Brischetto A Martinez-Campanario MC. Inflammatory macrophages reprogram to immunosuppression by reducing mitochondrial translation. Nat Commun. 2023;14(1):7471. doi: 10.1038/s41467-023-42277-4. 37978290 10.1038/s41467-023-42277-4 PMC10656499

[14] Ting KKY Yu P Dow R. Oxidized low-density lipoprotein accumulation suppresses glycolysis and attenuates the macrophage inflammatory response by diverting transcription from the HIF-1α to the Nrf2 pathway. J Immunol. 2023;211(10):1561-77. doi: 10.4049/jimmunol.2300293. 37756544 10.4049/jimmunol.2300293 PMC10873122

[15] Sieg SF Bazdar DA Zidar D. Highly oxidized low-density lipoprotein mediates activation of monocytes but does not confer interleukin-1β secretion nor interleukin-15 transpresentation function. Immunology. 2020;159(2):221-30. doi: 10.1111/imm.13142. 31663113 10.1111/imm.13142 PMC6954695

[16] Gibbs ET Lerner CA Watson MA. Site IQ in mitochondrial complex I generates S1QEL-sensitive superoxide/hydrogen peroxide in both the reverse and forward reactions. Biochem J. 2023;480(5):363-84. doi: 10.1042/BCJ20220611. 36862427 10.1042/BCJ20220611 PMC10212513

[17] Wong HS Monternier PA Brand MD. S1QELs suppress mitochondrial superoxide/hydrogen peroxide production from site IQ without inhibiting reverse electron flow through Complex I. Free Radic Biol Med. 2019;143:545-59. doi: 10.1016/j.freeradbiomed.2019.09.006. 31518685 10.1016/j.freeradbiomed.2019.09.006

[18] Grund EM Clarkson BDS Pucci S. Pentose phosphate pathway inhibition metabolically reprograms CD8+ T cells and disrupts CNS autoimmunity. JCI Insight. 2025;10(14):e184240. doi: 10.1172/jci.insight.184240. 40493395 10.1172/jci.insight.184240 PMC12288970

[19] Orekhov AN Oishi Y Nikiforov NG. Modified LDL particles activate inflammatory pathways in monocyte-derived macrophages: transcriptome analysis. Curr Pharm Des. 2018;24(26):3143-51. doi: 10.2174/1381612824666180911120039. 30205792 10.2174/1381612824666180911120039 PMC6302360

[20] Vendrov AE Lozhkin A Hayami T. Mitochondrial dysfunction and metabolic reprogramming induce macrophage pro-inflammatory phenotype switch and atherosclerosis progression in aging. Front Immunol. 2024;15:1410832. doi: 10.3389/fimmu.2024.1410832. 38975335 10.3389/fimmu.2024.1410832 PMC11224442

[21] Cai S Zhao M Zhou B. Mitochondrial dysfunction in macrophages promotes inflammation and suppresses repair after myocardial infarction. J Clin Invest. 2023;133(4):e159498. doi: 10.1172/JCI159498. 36480284 10.1172/JCI159498 PMC9927948

